# Supplementary material for: Indigenous microorganisms offset the benefits of growth and nutrition regulated by inoculated arbuscular mycorrhizal fungi for four pioneer herbs in karst soil
Source: PLoS One. 2022 Apr 25;17(4):e0266526. doi: 10.1371/journal.pone.0266526 (PMC9037916; doi:10.1371/journal.pone.0266526)
Supplement: Attachment — Submitted filename: PONE-D-21-40761(revised).pdf [file pone.0266526.s001.pdf]

PLOS ONE

Indigenous microorganisms relieved the benefits of growth and nutrition regulated by arbuscular mycorrhizal fungi for four pioneer herbs in karst soil

--Manuscript Draft--

**Manuscript Number:**	PONE-D-21-40761
**Article Type:**	Research Article
**Full Title:**	Indigenous microorganisms relieved the benefits of growth and nutrition regulated by arbuscular mycorrhizal fungi for four pioneer herbs in karst soil
**Short Title:**	Indigenous microorganisms relieved the benefits of growth and nutrition regulated by AM fungi for karst herbs
**Corresponding Author:**	Yuejun He, Ph.D<br>Guizhou University<br>Guiyang City, Guizhou Province CHINA
**Keywords:**	Arbuscular mycorrhizal fungi; Indigenous microorganisms; Nutrients; Karst plants
**Abstract:**	Arbuscular mycorrhizal (AM) fungi are beneficial soil microorganisms and inevitably interact with indigenous microorganisms, regulating plant growth and nutrient utilization in natural habitats. However, the role of indigenous microorganisms in affecting the growth and nutritional functions of plants regulated by AM fungi in karst ecosystem habitats remains unclear still today. In this experiment, the Gramineae species Setaria viridis vs. Arthraxon hispidus and the Compositae species Bidens pilosa vs. Bidens tripartita existing in the initial succession stage of the karst ecosystem were planted into different soil microbial conditions, including AM fungi soil (AMF), AM fungi interacting with indigenous microorganisms (AMI), and a control soil without AM fungi and indigenous microorganisms (CK). The plant biomass, nitrogen (N), and phosphorus (P) were measured; the effect size of different treatments on these variables of plant biomass and N and P were simultaneously calculated to assess plant respons. The results showed that AMF treatment differently enhanced plant biomass, N, and P in all species, but reduced the N/P ratio. The AMI treatment also significantly increased plant biomass, N and P, except for the S. viridis seedlings. However, in the effect size, the AM fungi effect on the plant growth and nutrition was greater than the interactive effect related to AM fungi interacting with indigenous microbes, and it indicates that the indigenous microorganisms relieved the AM benefits for the host plant. In addition, the plant biomass, N, and P were significantly different between A. hispidus and S. viridis of Gramineae, while non-significantly different for B. pilosa and B. tripartita of Compositae under AMF treatment. In conclusion, ~~we suggest that~~ the indigenous microorganisms relieved the benefits of AM fungi in biomass and nutrient accumulation for pioneer plants in the karst habitat.
**Order of Authors:**	Yan Sun
	Muhammud Umer
	Yuejun He
	Yun Guo
	Wenda Ren
	Xu Han
	Qing Li
	Bangli Wu
	Kaiping Shen
	Tingting Xia
	Lu Gao
	Jinting Li
	Xiaorun Hu

Powered by Editorial Manager® and ProduXion Manager® from Aries Systems Corporation

	Li Wang
	Lipeng Zang
	Shixiong Wang
**Additional Information:**	
**Question**	**Response**
**Financial Disclosure**<br><br>Enter a financial disclosure statement that describes the sources of funding for the work included in this submission. Review the submission guidelines for detailed requirements. View published research articles from *PLOS ONE* for specific examples.<br><br>This statement is required for submission and **will appear in the published article** if the submission is accepted. Please make sure it is accurate.<br><br>**Unfunded studies**<br>Enter: *The author(s) received no specific funding for this work.*<br><br>**Funded studies**<br>Enter a statement with the following details:<br>• Initials of the authors who received each award<br>• Grant numbers awarded to each author<br>• The full name of each funder<br>• URL of each funder website<br>• Did the sponsors or funders play any role in the study design, data collection and analysis, decision to publish, or preparation of the manuscript?<br>• **NO** - Include this sentence at the end of your statement: *The funders had no role in study design, data collection and analysis, decision to publish, or preparation of the manuscript.*<br>• **YES** - Specify the role(s) played.<br><br>* typeset	YES. This research was funded by the National Natural Science Foundation of China (NSFC: 31660156; 31360106), the Science and Technology Project of Guizhou Province ([2021] General-455; [2016] Supporting-2805), the Guizhou Hundred-level Innovative Talents Project (Qian-ke-he platform talents [2020] 6004), the First-class Disciplines Program on Ecology of Guizhou Province (GNYL[2017]007), the Talent-platform Program of Guizhou Province ([2017]5788; [2018]5781), theBasicResearchPrograminGuizhouProvince([2019]1060).
**Competing Interests**<br><br>Use the instructions below to enter a competing interest statement for this submission. On behalf of all authors, disclose any competing interests that could be perceived to bias this	NO. The authors have declared that no competing interests exist.

work—acknowledging all financial support and any other relevant financial or non-financial competing interests.<br><br>This statement is required for submission and **will appear in the published article** if the submission is accepted. Please make sure it is accurate and that any funding sources listed in your Funding Information later in the submission form are also declared in your Financial Disclosure statement.<br><br>View published research articles from *PLOS ONE* for specific examples.<br><br>**NO authors have competing interests**<br><br>Enter: *The authors have declared that no competing interests exist.*<br><br>**Authors with competing interests**<br><br>Enter competing interest details beginning with this statement:<br><br>*I have read the journal's policy and the authors of this manuscript have the following competing interests: [insert competing interests here]*<br><br>* typeset	
**Ethics Statement**<br><br>Enter an ethics statement for this submission. This statement is required if the study involved:<br><br>• Human participants<br>• Human specimens or tissue<br>• Vertebrate animals or cephalopods<br>• Vertebrate embryos or tissues<br>• Field research<br><br>Write "N/A" if the submission does not require an ethics statement.	N/A

General guidance is provided below. Consult the submission guidelines for detailed instructions. **Make sure that all information entered here is included in the Methods section of the manuscript.**<br><br>**Format for specific study types**<br><br>**Human Subject Research (involving human participants and/or tissue)**<br>• Give the name of the institutional review board or ethics committee that approved the study<br>• Include the approval number and/or a statement indicating approval of this research<br>• Indicate the form of consent obtained (written/oral) or the reason that consent was not obtained (e.g. the data were analyzed anonymously)<br><br>**Animal Research (involving vertebrate animals, embryos or tissues)**<br>• Provide the name of the Institutional Animal Care and Use Committee (IACUC) or other relevant ethics board that reviewed the study protocol, and indicate whether they approved this research or granted a formal waiver of ethical approval<br>• Include an approval number if one was obtained<br>• If the study involved *non-human primates*, add *additional details* about animal welfare and steps taken to ameliorate suffering<br>• If anesthesia, euthanasia, or any kind of animal sacrifice is part of the study, include briefly which substances and/or methods were applied<br><br>**Field Research**<br><br>Include the following details if this study involves the collection of plant, animal, or other materials from a natural setting:<br>• Field permit number<br>• Name of the institution or relevant body that granted permission	
**Data Availability**<br><br>Authors are required to make all data underlying the findings described fully	Yes - all data are fully available without restriction

Powered by Editorial Manager® and ProduXion Manager® from Aries Systems Corporation

available, without restriction, and from the time of publication. PLOS allows rare exceptions to address legal and ethical concerns. See the PLOS Data Policy and FAQ for detailed information.<br><br>A Data Availability Statement describing where the data can be found is required at submission. Your answers to this question constitute the Data Availability Statement and **will be published in the article**, if accepted.<br><br>**Important:** Stating 'data available on request from the author' is not sufficient. If your data are only available upon request, select 'No' for the first question and explain your exceptional situation in the text box.<br><br>Do the authors confirm that all data underlying the findings described in their manuscript are fully available without restriction?	
**Describe where the data may be found in full sentences. If you are copying our sample text, replace any instances of XXX with the appropriate details.**<br><br>• If the data are **held or will be held in a public repository**, include URLs, accession numbers or DOIs. If this information will only be available after acceptance, indicate this by ticking the box below. For example: *All XXX files are available from the XXX database (accession number(s) XXX, XXX.)*.<br>• If the data are all contained **within the manuscript and/or Supporting Information files**, enter the following: *All relevant data are within the manuscript and its Supporting Information files.*<br>• If neither of these applies but you are able to provide **details of access elsewhere**, with or without limitations, please do so. For example:<br><br>*Data cannot be shared publicly because of [XXX]. Data are available from the XXX Institutional Data Access / Ethics*	All relevant data are within the manuscript and its Supporting Information files.

Committee (contact via XXX) for researchers who meet the criteria for access to confidential data.<br><br>*The data underlying the results presented in the study are available from (include the name of the third party and contact information or URL).*<br>• This text is appropriate if the data are owned by a third party and authors do not have permission to share the data.<br><br>* typeset	
Additional data availability information:	

Powered by Editorial Manager® and ProduXion Manager® from Aries Systems Corporation

Manuscript Click here to access/download;Manuscript;manuscript.docx

Indigenous microorganisms relieved the benefits of growth and nutrition regulated by arbuscular mycorrhizal fungi for four pioneer herbs in karst soil

Yan Sun[1], Muhammud Umer[2], Yuejun He [1,2]*, Yun Guo[1], Wenda Ren[1], Xu Han[1], Qing Li[1], Bangli Wu[1], Kaiping Shen[1], Tingting Xia[1], Lu Gao[1], Jinting Li[1], Xiaorun Hu[1], Li Wang[1], Lipeng Zang[1], Shixiong Wang[1]

[1] Forestry College, Research Center of Forest Ecology, Guizhou University, Guiyang 550025, P. R. China

2. Institute for Forest Resources & Environment of Guizhou, Guizhou University, Guiyang 550025, P. R. China

*Corresponding author: Yuejun He

E-mail address: hyj1358@163.com

Abstract: Arbuscular mycorrhizal (AM) fungi are beneficial soil microorganisms and inevitably interact with indigenous microorganisms, regulating plant growth and nutrient utilization in natural habitats. However, the role of indigenous microorganisms in affecting the growth and nutritional functions of plants regulated by AM fungi in karst ecosystem habitats remains unclear still today. In this experiment, the Gramineae species *Setaria viridis* vs. *Arthraxon hispidus* and the Compositae species *Bidens pilosa* vs. *Bidens tripartita* existing in the initial succession stage of the karst ecosystem were planted into different soil microbial conditions, including AM fungi soil (*AMF*), AM fungi interacting with indigenous microorganisms (*AMI*), and a control soil without AM fungi and indigenous microorganisms (*CK*). The plant biomass, nitrogen (N), and phosphorus (P) were measured; the effect size of different treatments on these variables of plant biomass and N and P were simultaneously calculated to assess plant respons. The results showed that AMF treatment differently enhanced plant biomass, N, and P in all species, but reduced the N/P ratio. The AMI treatment also significantly increased plant biomass, N and P, except for the *S. viridis* seedlings. However, in the effect size, the AM fungi effect on the plant growth and nutrition was greater than the interactive effect related to AM fungi interacting with indigenous microbes, and it indicates that the indigenous microorganisms relieved the AM benefits for the host plant. In addition, the plant biomass, N, and P were significantly different between *A. hispidus* and *S. viridis* of Gramineae, while non-significantly different for *B. pilosa* and *B. tripartita* of Compositae under *AMF* treatment. In conclusion, we suggest that the indigenous microorganisms relieved the benefits of AM fungi in biomass and nutrient accumulation for pioneer plants in the karst habitat.

Keywords: Arbuscular mycorrhizal fungi; Indigenous microorganisms; Nutrients; Karst plants

1. Introduction

Karst ecosystem occupies approximately 7~12% of emerged land globally, mainly distributed in southwest China, and is characterized by high habitat heterogeneity and high vegetation

fragmentation [1] with high soil erosion, rocky desertification, and barren vegetation nutrient deficiency [2-7]. However, the karst vegetation retains a robust natural resilience even in harsh habitats [8,9]. Initially, herbaceous plants as the pioneers adapting to these areas, such as Gramineae and Compositae, have prominent advantages in resisting drought and barrenness and have fast growth with improved soil structure [10] in the initial succession stage ecosystem restoration [11,12]. In addition, soil microorganisms play an essential role in recovering degraded karst systems [13] through promoting the growth and nutrient uptake by plants [14,15] as well as improvement of soil nutrients, increasing their bioavailability [16]. Thus, soil microorganisms in karst vegetation restoration cannot be ignored, particularly arbuscular mycorrhizal (AM) fungi.

AM fungi as a soil functional microorganism can play a critical role in recovering degraded terrestrial ecosystems [17]. AM fungi formed a symbiotic relationship with 80% of terrestrial plants [18,19] and improved plant growth, nutrient accumulation [20,21], also enhancing drought stress tolerance [22], and maintained soil structure [23], e.g., Guo, He et al. (2021) [24] proposed that AM fungi differently affected the competitive ability of *Broussonetia papyrifera* and *Carpinus pubescens* on growth and nutrient accumulation; Xia, Wang et al. (2020) [25] also showed that AM fungi increased nutrients for host plants by regulating the morphological development of karst plant roots. In addition, Shi, Mickan et al. (2015) [26] also illustrated that AM fungi enhanced the biomass, N, and P content in shoots and roots of plants. Furthermore, AM fungi mycelium can transfer the photosynthetic carbohydrates from the host plants to the soil, which recruits soil microorganisms [27]. However, we know relatively little about how AM fungi regulate plant

growth and nutrient through interacting with indigenous microorganisms.

AM fungi via extensive extraradical hyphae interacting with indigenous microbial communities play a crucial role in plant growth in natural habitats [28,29]. AM fungi and bacteria are ubiquitous in natural soil [30]. Specifically, AM fungi regulate plant growth, and it is positively affected by cooperating with indigenous microorganisms [31,32] or negatively affected by competing with indigenous microorganisms [33,34]. Ortiz, Armada et al. (2015) [31] suggested that the combination of AM fungi and specific bacteria could promote plant growth by minimizing drought-related stress effects. Artursson, Finlay et al. (2006) [35] also proposed that the co-inoculation of AM fungi and phosphorus-solubilizing bacteria positively promotes plant nutrient absorption. In addition, some bacteria such as plant growth-promoting rhizobacteria could promote mycorrhizal fungal activity and establishment [36-38]; sometimes, such indigenous bacteria are called mycorrhiza helper bacteria [39]. In contrast, the competition phenomenon between AM fungi and bacteria has been widely reported [40]. Azcón-Aguilar and Barea (1997) [41] presented evidence of direct competition between AM fungi and indigenous microorganisms for photosynthetic products of the host plant in order to their growth. Indirectly, Doumbou, Hamby Salove et al. (2005) [42] proposed that many *Streptomyces* sp. could exude antifungal compounds, which indicated that they are fungal competitors under the appropriate environmental conditions. Therefore, it is a complex relationship among AM fungi and indigenous microorganisms.

In summary, AM fungi play an important role in improving plant growth and nutrient absorption. However, AM fungi inevitably interact with indigenous microorganisms in the vegetation

restoration of the karst-degraded ecosystem. It remains unclear how indigenous microorganisms affect the benefits of growth and nutrition regulated by AM fungi for plants in karst soils. Because of the complexity and uncertainty of the interaction between AM fungi with indigenous microorganisms, it is necessary to assess the effect size of AM fungi and indigenous microorganisms, and their interaction, on plant growth and nutrition. The aim was to clarify how indigenous microorganisms affect the benefits of growth and nutrition regulated by AM fungi for plants in karst soils. Thus, an experiment was conducted on four pioneer herb plants of Gramineae *Setaria viridis* vs. *Arthraxon hispidus* and Compositae *Bidens pilosa* vs *Bidens tripartita* existing in the initial study succession stage of the karst ecosystem, cultivated into different microbial soil, including arbuscular mycorrhizal fungi accompanying with indigenous microorganisms. We hypothesize that: (1) AM fungi can promote the growth and the nutrient accumulation of four pioneer plants in karst (H1); (2) Indigenous microorganisms relieved the benefits of AM fungi on plant growth and nutrient accumulation (H2).

2. Materials and Methods

2.1. Experiment treatments

A potting experiment was conducted by using four herb species: *Setaria viridis*, *Arthraxon hispidus*, *Bidens pilosa*, and *Bidens tripartita* in polypropylene plastic pots in a greenhouse of Guizhou University in Guiyang, China (E: 106°22′ E; N: 29°49′ N; 1,120 m.a.s.l.). Three different soil microbial conditions were created for exploring the interaction of AM fungi with indigenous soil microbes in the regulation of plant growth and nutrient utilization, including the arbuscular

mycorrhizal fungi soil by inoculating AM fungi into sterilized soil (*AMF* treatment), the soil of arbuscular mycorrhizal fungi interacting with indigenous microorganisms by inoculating AM fungi into soil that contained indigenous soil microorganism from natural condition (*AMI* treatment), and the control soil by removing microorganism with sterilization (*CK* treatment). In the beginning, limestone soil was collected from a typical karst habitat, from which approximately two-thirds of the soil was used for sterilization at 126 ℃, 0.14 Mpa for one hour to eliminate microbes, and one-third of the soil was retained for further experiments. Subsequently, a 2.5 kg soil subsample of the sterilized or unsterilized soil was put into each polypropylene plastic pot (180 mm × 160 mm, diameter × height). Five seeds of *Setaria viridis*, *Arthraxon hispidus*, *Bidens pilosa*, and *Bidens tripartita* were disinfected with a 10% H_2O_2 solution for 10 minutes and repeatedly washed with sterile water, and sown in each pot. After sowing seeds in each pot, seeds were covered with 200 g of the respective soil according to the ~~treatment, and thus it~~ promoted the germination rate. In addition, the sterilized soil was inoculated with 10 g *Glomus mosseae* as the *AMF* treatment, and the original soil from field habitat was inoculated with 10 g of *Glomus mosseae* as the *AMI* treatment, indicating the AM fungi interacting with the indigenous microbes in this experiment. Especially, a 10 g of sterilized inoculum of *Glomus mosseae* was taken into the *CK* treatment, along with 10 ml of the filtrate by weighing 10 g of *Glomus mosseae* inoculum with sterile water using a double-layer filter paper. The aim was to maintain the consistency of microflora except for the targeted fungus *Glomus mosseae* corresponding to *AMF* treatment. There is mutual control between two of three treatments: the AM fungi effect may be obtained by

comparing *AMF* with *CK* treatment; similarly, the interactive effect relating to AM fungi with indigenous microbes may be obtained by comparing *AMI* with *CK* treatment, and the indigenous microbial effect relating to AM fungi may be obtained by comparing *AMI* with *AMF* treatment. Of course, we had to admit that the unsterilized soil probably had native AM fungi under *AMI* treatment, even the targeted species *Glomus mosseae*. However, it was sure that the *Glomus mosseae* inoculum interacted with native AMF species and indigenous microorganisms; further, they jointly affected plants and soil for growth and nutrition when comparing *AMI* with *AMF*. All treatments were replicated five times, and four plant species contained 60 pots.

The physicochemical properties of limestone soil (per kg) were measured by the methods from Tan (2005) [43], the PH 8.2, total nitrogen (TN) 0.622 g, alkaline hydrolysis nitrogen (AN) 0.315 g, total phosphorus (TP) 1.274 g, available phosphorus (AP) 0.163 g, total potassium (TK) 37.79 g, and available potassium (AK) 0.532 g. All plant seeds were also collected from the same karst habitat used to collect soil. According to the primary field survey, these plants are successive pioneer species of karst communities as the herbaceous stage, which generally coexist in the same habitat as the main Gramineae and Compositae. Three weeks after seeds germination, only two seedlings were retained in the pot and cultured for five months, and all growing seedlings were watered one time per day for maintaining field capacity, then harvested to determine the biomass, N, and P concentrations. The *Glomus mosseae* inoculum was initially purchased from the Institute of Nutrition Resources, Beijing Academy of Agricultural and Forestry Sciences (NO.BGA0046). It was propagated for 4 months with *Trifolium repens* including spores (above 100 spores per gram

of soil), mycelium, and root pieces in the laboratory of Guizhou University before the start of the experiment.

2.2. Determinations of the root mycorrhizal colonization, biomass, and the accumulation of nitrogen and phosphorus

The grid line-intersect method determined the root mycorrhizal colonization rate [44]. The biomass of *S. viridis*, *A. hispidus*, *B. pilosa,* and *B. tripartita* were respectively determined by weighing tissue of root, stem, and leaves after drying at 80℃ for 24 h. The nitrogen and phosphorus concentrations in plant tissue were determined by the traditional Kjeldahl method and the Molybdenum-antimony anti-colorimetric method, respectively [45]. Additionally, the accumulations of nitrogen and phosphorus were calculated using nutrient concentration multiplied by biomass.

2.3. Calculation of effect size

The effect size was calculated using the response ratio (lnR) of treatment groups to the control groups plant biomass referred from the proposition of [46] regarding the plant response mycorrhizal fungi. Due to the mutual control between two of three treatments in this experiment, the mutual effect size was calculated by the *AMF vs. CK* for AM fungi effect (*AME*) related to without AM fungi, *AMI vs. CK* for the interactive effect related to AM fungi interacting with indigenous microbes (*AIE*), and the *AMI vs. AMF* for the indigenous microbial effect related to AM fungi (*IME*). Therefore, the modified method was adopted according to Hoeksema, Chaudhary et al. (2010) [46] and Hedges, Gurevitch et al. (1999) [47] as follows:

$$\ln R = \ln(Xt / Xc)$$

Where Xt and Xc represent the biomass or nutrient accumulation of the plant in the values of the treatment group and control group, respectively, values> 0 indicate positive effects promoting plant growth or nutrient accumulation, values < 0 indicate negative effects suppressing plant growth or nutrient accumulation.

2.4. Statistical analyses

All of the statistical analyses were performed through SPSS 25.0 software. Two-way ANOVA was applied for assessing the effects of plant species (Ps; *Setaria viridis* vs. *Arthraxon hispidus* vs. *Bidens pilosa* vs. *Bidens tripartita*), soil microbial treatments (Ms; *AMI* vs. *AMF* vs. *CK*), and their interactions (Ms×Ps) on plant biomass, nitrogen accumulation, and phosphorus accumulation, N/P ratio and effect size by the ln*R*. The least significant difference (LSD) test was applied to compare significant differences in root mycorrhizal colonization, biomass, nitrogen, and phosphorus accumulations, and N/P ratio with effect size by the ln*R* among the three different conditions of soil microbial treatments with *AMI*, *AMF*, and *CK* or four plant species of *Setaria viridis* and *Arthraxon hispidus* and *Bidens pilosa* and *Bidens tripartita* at $P \leq 0.05$. All graphs were drawn on Origin 2018.

3. Results

3.1. Root mycorrhizal colonization of four plant species under different microbial treatments

A non-significant *AMI* > *AMF* of root mycorrhizal colonization was found in the four species; however, the root mycorrhizal colonization of *CK* treatment was zero; meanwhile, the AM fungus spore and mycelium was not discovered under *CK* soil substrate via the microscopic detection (**Table 1**). The root mycorrhizal colonization of *B. pilosa* and *B. tripartita* were significantly greater than that of *A. hispidus* and *S. viridis,* respectively, while for *A. hispidus,* it was also greater than *S. viridis*. Besides, there was no significant difference in root mycorrhizal colonization of *B. pilosa* and *B. tripartita* under *AMI* and *AMF* treatments (**Table 1)**. These results indicate root mycorrhizal colonization is species differences, and it provides evidence for host preferences of AM fungal.

3.2. Biomass and its response ratio of four plant species under different microbial treatments

The soil microbial condition treatments (Ms) significantly affected biomass (**Table 2**). Significantly *AMF* > *AMI* > *CK* of biomass were observed in *A. hispidus*, *B. pilosa,* and *B. tripartita* seedlings except for *S. viridis*. Plant biomass was increased by AM fungus when comparing *AMF* with *CK* and *AMI* with *CK*, respectively. However, the plant biomass under *AMI* was significantly lower than under *AMF* (**Fig. 1A**). The plant species (Ps) also significantly affected individual biomass (**Table 2**). Under *AMF* and *CK* treatments, the biomass of *A. hispidus* was significantly greater than the other three species, while the biomass of *S. viridis* was significantly lower than the other three species under *AMF* and *AMI* treatments. In addition, there was a non-significant difference in biomass observed between *B. pilosa* and *B. tripartita* seedlings

under any soil microbial condition treatments (**Fig. 1A**). Meanwhile, the interaction of Ms×Ps significantly affected the individual biomass for the four species (**Table 2**). The results revealed that *AMF* and *AMI* treatments significantly increased the biomass accumulation of four karst pioneer species. Meanwhile, the biomass was significantly different between *A. hispidus* and *S.viridis* of Gramineae, except for *B. pilosa* and *B. tripartita* under *AMF*.

Similarly, the soil microbial condition treatments (Ms), the plant species (Ps), and their interaction significantly affected the response ratio of biomass ($\ln R_{Biomass}$) (**Table 2**). On the one hand, a positive effect ($\ln R_{Biomass} > 0$) of biomass was observed in the four species under *AME* and *AIE* conditions except for *S. viridis* in *AIE* (**Fig. 1B**). However, a significant *AME* > *AIE* was observed in $\ln R_{Biomass}$, indicating that AM fungus was beneficial for plant biomass, but the positive effect was decreased when AM fungi interacted with indigenous microorganisms. On the other hand, a negative effect ($\ln R_{Biomass} < 0$) was shown in the *IME* condition (**Fig. 1B**), indicating that indigenous microorganisms relieved the AM fungi promotion in plant growth. Precisely, the results indicated that AM fungi significantly increased the biomass accumulation of four karst pioneer species, but the $\ln R_{Biomass}$ reduction by comparing *AIE* to *AME* specified that the indigenous microorganisms relieved the benefits of AM fungi on plant biomass.

3.3. Nitrogen accumulation and its response ratio of four plant species under different microbial treatments

The soil microbial condition treatments (Ms) significantly affected N accumulation (**Table 2**). Significantly *AMF* > *AMI* > *CK* of N accumulation were shown in *A. hispidus*, *B. pilosa,* and *B.*

tripartita seedlings, except for *S. viridis*. Specifically, the N accumulation was enhanced by AM fungus when comparing *AMF* with *CK* and *AMI* with *CK*, respectively. At the same time, N accumulation under *AMI* was significantly lower than under *AMF* (**Fig. 2A**). The plant species (Ps) also significantly affected N accumulation (**Table 2**). Under *AMF* and *AMI* treatments, N accumulation in *S. viridis* was significantly lower than the other three species, while N accumulation in *A. hispidus* was significantly greater than the other three species under *CK* treatment. Moreover, there was a non-significant difference in N accumulation between *B. pilosa* and *B. tripartita* seedlings under any soil microbial condition treatments (**Fig. 2A**). Furthermore, the interaction of Ms×Ps significantly affected the N accumulation for the four species (**Table 2**). These results showed that *AMF* and *AMI* treatments significantly increased the N accumulation of four karst pioneer species. Meanwhile, N accumulation was significantly different between *A. hispidus* and *S. viridis*, but not for *B. pilosa* and *B. tripartita* under *AMF*.

Similarly, the soil microbial condition treatments (Ms), the plant species (Ps), and their interaction significantly affected the response ratio of N ($\ln R_N$) (**Table 2**). One side has a positive effect ($\ln R_N > 0$) of N was observed in four species under *AME* and *AIE* conditions except for *S. viridis* in *AIE* (**Fig. 2B**). However, a significant *AME* > *AIE* was observed in $\ln R_N$, indicating that AM fungus was beneficial for plant N accumulation, but the positive effect was decreased when AM fungi interacted with indigenous microorganisms. Another side has a negative effect ($\ln R_N < 0$) obtainable in the *IME* condition (**Fig. 2B**), indicating that indigenous microorganisms relieved the AM fungi promotion in N accumulation. Overall, the results directed that AM fungi significantly

increased the N accumulation of four karst pioneer species, while the lnR_N reduction by comparing *AIE* to *AME* specified that the indigenous microorganisms relieved the benefits of AM fungi on N accumulation.

3.4. Phosphorous accumulation and its response ratio of four plant species under different microbial treatments

The soil microbial condition treatments (Ms) significantly affected P accumulation (**Table 3**). Significantly *AMF* > *AMI* > *CK* of P accumulation was admissible in four species. Unambiguously, AM fungus enhanced P accumulation when comparing *AMF* with *CK* and *AMI* with *CK*; but the P accumulation under *AMI* was significantly lower than under *AMF* (**Fig. 3A**). The plant species (Ps) also significantly affected P accumulation (**Table 3**). Under *AMF* and *AIE* treatments, the P accumulation in *S. viridis* was significantly lower than the other three species, while the P accumulation of *A. hispidus* was significantly greater than the other three species under *CK* treatments. In addition, there was no significant difference in P accumulation between *B. pilosa* and *B. tripartita* seedlings under any microbial condition soil treatments (**Fig. 3A**). Meanwhile, the interaction of Ms×Ps significantly affected the P accumulation for four species (**Table 3**). This points out that *AMF* and *AMI* treatments significantly increased the P accumulation of four karst pioneer species. Meanwhile, P accumulation was significantly different between *A. hispidus* and *S. viridis* of Gramineae, except for *B. pilosa* and *B. tripartita* of Compositae under *AMF*.

Likewise, the soil microbial condition treatments (Ms), the plant species (Ps), and their interaction significantly affected the response ratio of P (lnR_P) (**Table 3**). Alternatively, it has a positive effect

($\ln R_P > 0$) of P on four species under *AME* and *AIE* conditions; however, a significant *AME* > *AIE* was observed in $\ln R_P$, indicating that AM fungus was beneficial for plant P accumulation, but the positive effect was decreased when AM fungi interacted with indigenous microorganisms (**Fig. 3B**). It also has a negative effect ($\ln R_P < 0$) in the *IME* condition and depicts that indigenous microorganisms relieved the AM fungi promotion in P accumulation. Therefore, the results consolidated that AM fungi significantly increased P accumulation of four karst pioneer species, then the $\ln R_P$ reduction by comparing *AIE* to *AME* designated that the indigenous microorganisms relieved the benefits of AM fungi on P accumulation.

3.5. N/P ratio and its response ratio of four plant species under different microbial treatments

The soil microbial condition treatments (Ms) significantly affected the N/P ratio **(Table 3)**, significantly greater N/P ratio between plant species ranked as the *CK* > *AMF* ≈ *AMI* for *S. viridis*, the *AMI* > *CK* ≈ *AMF* for *A. hispidus*, the *AMI* > *AMF* > *CK* for *B. Pilosa*, and *CK* ≈*AMI* > *AMF* for *B. tripartita* (**Fig. 4A**). The plant species (Ps) also significantly affected the N/P ratio (**Table 3**), and the N/P ratio for four plants showed species differences under different soil microbial treatments. Explicitly, there was a non-significant difference in the N/P ratio of the four species under *AMF* treatments. Under *AMI* treatments, the N/P ratio of *A. hispidus* and *B. tripartita* were significantly greater than *S. viridis* and *B. pilosa*, respectively. In the interim, the N/P ratio of the *B. pilosa* was greater than *S. viridis* seedlings. Under *CK* treatment, the N/P ratio of *B. tripartita* was significantly greater than the other three species, while the N/P ratio of *B. pilosa* was

significantly lower than the other three species (**Fig. 4A**). Likewise, the interaction of Ms×Ps significantly affected the N/P ratio for four species (**Table 3**). Therefore, AM fungi significantly reduced the N/P ratio of four species. Equally, the soil microbial condition treatments (Ms), the plant species (Ps), and their interaction significantly affected the response ratio of N/P ($\ln R_{N/P}$) (**Table 3**). Three microbial effects resulted in negative N/P of *S. viridis* responses ($\ln R_{N/P} < 0$), while three microbial effects resulted in positive N/P of *B. Pilosa* response ($\ln R_{N/P} > 0$) (**Fig. 4B**). For *A. hispidus*, a positive effect was observed in *IME* and *AIE*, while a negative effect was found in *AME*. For *B. tripartita*, a positive effect was observed in *IME*, while a negative effect was found in *AME* and *AIE*. Overall, AM fungi significantly reduced the N/P ratio for the four-karst pioneer species, portraying that the AM fungi alleviate P limitation and promote plant growth in karst areas with low P.

4. Discussion

AM fungi significantly increased biomass and accumulation of N and P for the four-karst pioneer species revealed through this experiment, which specified positive influences of inoculation of AM fungi (**Fig. 1A, 2A, 3A**). Consistently, the positive influence of AM fungi inoculation on host plant growth and nutrient accumulation was also observed in some previous studies [48,49]. For instance, He, Jiang et al. (2017) [20] proposed that AM fungi enhanced plant growth and nutrient absorption of *B. papyrifera* and *B. pilosa* in karst soil and can compare with our results in AM fungi significantly increasing biomass and accumulation of N and P for the four plants. There are two main pathways that AM fungi promote plant growth and nutrient accumulation. One is that AM fungi can extend the absorbing network beyond the rhizosphere nutrient depletion region, and absorb a larger volume of soil mineral nutrients, thereby improving the ability of plants to obtain

nutrients [50] and ultimately benefiting plant growth [51-53]. For example, the poor mobility of phosphate ions in soils [54] limits plant growth due to forming a rhizosphere phosphorus-depletion region, and AM fungi hyphae can cross the zone to assist the host plant to absorb nutrients from a distance [55]. Our findings were similar to those of Shao, Hu et al. (2021) [56], who presented that AM fungi could promote the P uptake of plants in acidic soils with severe limitation of P. Another is that AM fungi can secrete organic acids and soil enzymes to dissolve the insoluble nutrients and mineralize the organic nutrient [57-59], thereby promoting the availability of soil nutrients [60]. Elbon and Whalen (2014) [61] illustrated that AM fungi could increase the plant-available P concentration by secreting organic acids and phosphatase enzymes. Therefore, AM fungi facilitated the growth and nutrient accumulation of four karst pioneer plants, which can verify the hypothesis of H1. However, the specific mechanism of AM fungi affecting nutrient accumulation of karst pioneer species needs to be explored further.

The N/P ratio can predict plant nutrient restrictions [62]. A low N/P ratio (< 14) indicates N limitation, whereas a high N/P ratio (> 16) indicates P limitation, and both N and P limit plant growth when the N/P ratio is between 14 and 16 [63]. In our experiment, the N/P of four species were greater than 16 under *AMI* and *CK* treatments, except for *S. viridis* under *AMI* and *B. pilosa* under *CK*, showing that plant growth was mainly limited by phosphorus in karst soil. However, the N/P ratio of the four species significantly decreased under *AMF* treatments compared with *AMI* and *CK* treatments for a whole (**Fig. 4A**). AM fungi reduced the N/P ratio of seedlings, representing that AM fungus is more effective in assisting plants to obtain P than N by alleviating P limitation. These results were similar to those of Shen, Cornelissen et al. (2020) [64], who suggested that AM fungi alleviated the P limitation of plants via the mycorrhizal network in low-

P karst soils. Consequently, the AM fungi play a vital role in alleviating the nutritional limitation of nutrient-deficient karst soils.

AM fungi enhanced four plants' biomass, N, and P accumulation differently. Which was more beneficial for *A. hispidus*, *B. pilosa*, and *B. tripartita* seedlings in biomass and nutrient accumulation than *S. viridis* seedlings (**Fig. 1A, 2A, 3A**), demonstrating that for different species, AM fungi play different roles in promoting host biomass and nutrient accumulation. Besides, the mycorrhizal colonization of *A. hispidus*, *B. pilosa*, and *B. tripartita* was significantly higher than *S. viridis* (**Table 1**), it was well proof of the different roles of AM fungi on different species, and these differences reflected that AM fungi had the selectivity for host plants. AM fungi showed host-specific growth response [65] and induced differential growth responses in host plant species [66]. It was similar to the research conducted by Liu and Wang (2003) [67], who proposed that *Nicotiana tabacum* was a more favorable host plant for *Glomus constrictum* and *Glomus multicaule*, compared to the other hosts. In addition, the host selectivity of AM fungi may be related to plant taxonomic levels of species, family, and genera [6]. In this study, the biomass and nutrients of N and P were significantly different between *A. hispidus* and *S. viridis* of Gramineae, except for *B. pilosa* and *B. tripartita* of Compositae under *AMF* (**Fig. 1A, 2A, 3A**). The possible explanation is that *A. hispidus* and *S. viridis* are of the same family but different genera, while *B. pilosa* and *B. tripartita* have a common family and genera [68,69] with identical root configurations, which might explain the similarity in AM fungi selection. Therefore, AM fungi are crucial for plant growth and nutrient utilization, but the mutual selection between AM fungi and

host plants cannot be ignored. The specific mechanism of selective plant-AMF combinations of karst pioneer species needs to be further explored.

In this experiment, the positive effects of AM fungi (*AME*) and interactive effect related to AM fungi interacting with indigenous microbes (*AIE*) on the plant growth and nutrient accumulation ranked as *AIE* < *AME* for a whole, while the negative effect of indigenous microbial related to AM fungi (*IME*) was found on plant growth and nutrient accumulation (**Fig. 1B, 2B, 3B**). It seems to imply that the indigenous microorganisms relieved the benefits of AM fungi on plant growth and nutrient accumulation, signifying a negative relationship between AM fungi and indigenous microorganisms. Previous studies have demonstrated that AM fungi interact with a wide variety of indigenous microorganisms [70,71]. Meanwhile, AM fungi regulated plant growth positively affected by cooperating with indigenous microorganisms [32] or negatively affected by competing with indigenous microorganisms [34], which depended on the species of indigenous microorganisms that interact with AM fungi [72-74]. Positively, Mortimer, Le Roux et al. (2012) [75] presented a synergistic relationship between AM fungi and nitrogen-fixing bacteria showing additive benefits for the growth and nutrient accumulation in the *Acacia cyclops*. In addition, Artursson, Finlay et al. (2006) [35] illustrated that the plant growth-promoting rhizobacteria (PGPR) could enhance the activity of AM during a symbiotic relationship with the host plant due to the stimulatory effects of PGPR on AM growth [76]. Negatively, Miransari (2011) [77] proposed that some bacteria in the rhizosphere would compete for resources with AM fungi or inhibit the activity of AM fungi, thereby affecting plant growth. In this study, AM fungi positively affected

plant growth and nutrient accumulation, but indigenous microorganisms reduced this effect, indicating a negative relationship between AM fungi and indigenous microorganisms. This is possibly caused by the competition between AM fungi and indigenous microorganisms, mainly including two sides. One side is interference competition, meaning that some microbes directly inhibit the function of AM fungi via exuding allelochemical substances [78] and bacterial antibiotics [79,80]. For example, Doumbou, Hamby Salove et al. (2005) [42] proposed that numerous streptomycetes could exude antifungal compounds, thereby inhibiting the function of AM fungi under certain environmental conditions. The other side is resources, and ecological niches competition, which was proposed by Leigh, Fitter et al. (2011) [81], who proposed that there was the resource competition for decomposition products between AM fungi and bacteria thereby resulting in an antagonistic relationship between them. Niwa, Koyama et al. (2018) [82] suggested that the fungus inoculum mainly competed with the indigenous fungi, probably because their life-history strategy was identical to the inoculum fungus. These results perhaps can explain why the indigenous microorganisms relieved the benefits of AM fungi on plant growth and nutrient accumulation, which was consistent with Biró, Köves-Péchy et al. (2000) [83], who found the indigenous microflora greatly reduced the functioning of the mycorrhizal inoculum. Collectively, indigenous microorganisms relieved the benefits of AM fungi in this study, which illustrated the interactions between AM fungi and indigenous microorganisms in karst areas should be mainly a negative relationship, it verified the hypothesis of H2 that indigenous microorganisms relieved the benefits of AM fungi on plant growth and nutrient accumulation. However, the specific mechanism

of the negative relationship between specific microorganisms and AM fungi in karst soil remains to be further studied.

5. Conclusions

In this experiment, AM fungi differently facilitated four plant accumulations on the biomass, N, and P; however, it reduced the N/P ratio. The interaction of AM fungi with indigenous microorganisms also significantly increased plant biomass, N, and P accumulation, except for *S. viridis* seedlings, while the indigenous microorganisms relieved the benefits of AM fungi for host plants. In addition, the biomass and nutrients of N and P were significantly different between *A. hispidus* and *S. viridis* of Gramineae, but not for *B. pilosa* and *B. tripartita* of Compositae under *AMF*. Finally, we can say that the indigenous microorganisms relieved the benefits of growth and nutrition regulated by arbuscular mycorrhizal fungi for pioneer plants in karst soil.

Acknowledgments

We thank Xinyang Xu for helping in this experiment. We are grateful to the Institute of Nutrition Resources, Beijing Academy of Agricultural and Forestry Sciences for providing *Glomus mosseae* (NO.BGA0046) for use in our experiments.

References

1. Nie YP, Chen HS, Wang KL, and Ding YL. Rooting characteristics of two widely distributed woody plant species growing in different karst habitats of southwest China. Plant Ecol. 2014;215(10):1099-1109. https://doi.org/10.1007/s11258-014-0369-0.
2. Liu Y, Liu C, Rubinato M, Guo K, Zhou J, and Cui M. An Assessment of Soil's Nutrient Deficiencies and Their Influence on the Restoration of Degraded Karst Vegetation in Southwest China. Forests. 2020;11(8):797. https://doi.org/10.3390/f11080797.
3. Peng T and Wang SJ. Effects of land use, land cover and rainfall regimes on the surface runoff and soil loss on karst slopes in southwest China. Catena. 2012;90(1):53-62. https://doi.org/10.1016/j.catena.2011.11.001.
4. Jiang Z, Lian Y, and Qin X. Rocky desertification in Southwest China: Impacts, causes, and restoration. Earth-Sci. Rev. 2014;132:1-12. https://doi.org/10.1016/j.earscirev.2014.01.005.
5. Green SM, Dungait JAJ, Tu C, Buss HL, Sanderson N, Hawkes SJ, et al. Soil functions and ecosystem services research in the Chinese karst Critical Zone. Chem. Geol. 2019;527. https://doi.org/10.1016/j.chemgeo.2019.03.018.
6. Yang H, Zang Y, Yuan Y, Tang J, and Chen X. Selectivity by host plants affects the distribution of arbuscular mycorrhizal fungi: evidence from ITS rDNA sequence metadata. BMC Evol. Biol. 2012;12(1):1-13. https://doi.org/doi:10.1186/1471-2148-12-50
7. Hartmann A, Goldscheider N, Wagener T, Lange J, and Weiler M. Karst water resources in a changing world: Review of hydrological modeling approaches. Rev. Geophys. 2014;52(3):218-242. https://doi.org/10.1002/2013rg000443.
8. Zeng F, Peng W, Song T, Wang K, Wu H, Song X, et al. Changes in vegetation after 22 years' natural restoration in the Karst disturbed area in northwestern Guangxi, China. Acta Ecol. Sin. 2007;27(12):5110-5119. https://doi.org/10.1016/s1872-2032(08)60016-5.
9. Pang D, Cao J, Dan X, Guan Y, Peng X, Cui M, et al. Recovery approach affects soil quality in fragile karst ecosystems of southwest China: Implications for vegetation restoration. Ecol. Eng. 2018;123:151-160. https://doi.org/10.1016/j.ecoleng.2018.09.001.
10. Long ZF, Tang CB, and Yang YC. Discussion on the effect of herbage in the comprehensive administration and the sustainable development in the karst area. Guizhou Agricultural Sciences. 2005;33(Z1):69-71.
11. Cao J, Yuan D, Tong L, Azim M, Yang H, and Huang F. An overview of karst ecosystem in Southwest China: current state and future management. J. Res. Ecol. 2015;6(4):247-256. https://doi.org/10.5814/j.issn.1674-764x.2015.04.008.
12. Anding L. The Composition and Structural Feature of Plant Community in Different Karst Stony Desertification Areas. Appl. Ecol. Environ. Res. 2017;15(4):1167-1183. https://doi.org/10.15666/aeer/1504_11671183.
13. Chen X, Su Y, He X, Wei Y, Wei W, and Wu J. Soil bacterial community composition and diversity respond to cultivation in Karst ecosystems. World J. Microbiol. Biotechnol. 2012;28(1):205-13. https://doi.org/10.1007/s11274-011-0809-0.
14. Richardson AE and Simpson RJ. Soil Microorganisms Mediating Phosphorus Availability. Plant Physiol. 2011;156(3):989-996. https://doi.org/doi:10.2307/41435012
15. Wang X, Yan B, Fan B, Shi L, and Liu G. Temperature and soil microorganisms interact to affect Dodonaea viscosa growth on mountainsides. Plant Ecol. 2018;219(7):759-774. https://doi.org/10.1007/s11258-018-0832-4.

16. Liu WX, Xu WH, Han Y, Wang CH, and Wan SQ. Responses of microbial biomass and respiration of soil to topography, burning, and nitrogen fertilization in a temperate steppe. Biol. Fert. Soils. 2007;44(2):259-268. https://doi.org/10.1007/s00374-007-0198-6.
17. Johnson NC, Wilson GW, Bowker MA, Wilson JA, and Miller RM. Resource limitation is a driver of local adaptation in mycorrhizal symbioses. P. Natl. Acad. Sci. USA. 2010;107(5):2093-8. https://doi.org/10.1073/pnas.0906710107.
18. Smith SE and Read DJ. Mycorrhizal Symbiosis. Q. Rev. Biol. 2008;3(3):273-281. https://doi.org/10.1097/00010694-198403000-00011.
19. Kardol P and Wardle DA. How understanding aboveground-belowground linkages can assist restoration ecology. Trends Ecol. Evol. 2010;25(11):670-9. https://doi.org/10.1016/j.tree.2010.09.001.
20. He YJ, Jiang CH, Yang H, Wang YJ, and Zhong ZC. Arbuscular mycorrhizal fungal composition affects the growth and nutrient acquisition of two plants from a karst area. Sains Malays. . 2017;46(10):1701-1708. https://doi.org/10.17576/jsm-2017-4610-05.
21. Ferrol N, Azcon-Aguilar C, and Perez-Tienda J. Review: Arbuscular mycorrhizas as key players in sustainable plant phosphorus acquisition: An overview on the mechanisms involved. Plant Sci. 2019;280:441-447. https://doi.org/10.1016/j.plantsci.2018.11.011.
22. Auge RM, Toler HD, and Saxton AM. Arbuscular mycorrhizal symbiosis alters stomatal conductance of host plants more under drought than under amply watered conditions: a meta-analysis. Mycorrhiza. 2015;25(1):13-24. https://doi.org/10.1007/s00572-014-0585-4.
23. Rillig MC and Mummey DL. Mycorrhizas and soil structure. New Phytol. 2006;171(1):41-53. https://doi.org/10.1111/j.1469-8137.2006.01750.x.
24. Guo Y, He YJ, Wu BL, Lin Y, He MH, Han X, et al. The interspecific competition presents greater nutrient facilitation compared to intraspecific competition through AM fungi interacting with litter for two host plants in karst soil. J. Plant Ecol. 2021. https://doi.org/10.1093/jpe/rtab110.
25. Xia T, Wang Y, He Y, Wu C, Shen K, Tan Q, et al. An invasive plant experiences greater benefits of root morphology from enhancing nutrient competition associated with arbuscular mycorrhizae in karst soil than a native plant. PLoS One. 2020;15(6):e0234410. https://doi.org/10.1371/journal.pone.0234410.
26. Shi Z, Mickan B, Feng G, and Chen Y. Arbuscular mycorrhizal fungi improved plant growth and nutrient acquisition of desert ephemeral Plantago minuta under variable soil water conditions. J. Arid Land. 2015;7(3):414-420. https://doi.org/10.1007/s40333-014-0046-0
27. Kaiser C, Kilburn MR, Clode PL, Fuchslueger L, Koranda M, Cliff JB, et al. Exploring the transfer of recent plant photosynthates to soil microbes: mycorrhizal pathway vs direct root exudation. New Phytol. 2015;205(4):1537-1551. https://doi.org/doi.org/10.1111/nph.13138.
28. Micallef SA, Shiaris MP, and Colón-Carmona A. Influence of Arabidopsis thaliana accessions on rhizobacterial communities and natural variation in root exudates. J. Exp. Bot. 2009;60(6):1729-1742. https://doi.org/10.1093/jxb/erp053.
29. Gahan J and Schmalenberger A. Arbuscular mycorrhizal hyphae in grassland select for a diverse and abundant hyphospheric bacterial community involved in sulfonate desulfurization. Appl. Soil Ecol. . 2015;89:113-121. https://doi.org/10.1016/j.apsoil.2014.12.008.
30. Marulanda A, Barea JM, and Azcon R. An indigenous drought-tolerant strain of Glomus intraradices associated with a native bacterium improves water transport and root development in Retama sphaerocarpa. Microb. Ecol.

2006;52(4):670-678. https://doi.org/10.1007/s00248-006-9078-0.

31. Ortiz N, Armada E, Duque E, Roldán A, and Azcón R. Contribution of arbuscular mycorrhizal fungi and/or bacteria to enhancing plant drought tolerance under natural soil conditions: effectiveness of autochthonous or allochthonous strains. J. Plant Physiol. . 2015;174:87-96. https://doi.org/10.1016/j.jplph.2014.08.019
32. Frey-Klett P and Garbaye J. Mycorrhiza helper bacteria: a promising model for the genomic analysis of fungal-bacterial interactions. New Phytol. 2005;168(1):4-8. https://doi.org/10.1111/j.1469-8137.2005.01553.x.
33. Vigo C, Norman J, and Hooker J. Biocontrol of the pathogen Phytophthora parasitica by arbuscular mycorrhizal fungi is a consequence of effects on infection loci. Plant Pathol. 2000;49(4):509-514. https://doi.org/10.1046/j.1365-3059.2000.00473.x.
34. Xiao Y, Zhao Z, Chen L, and Li Y. Arbuscular mycorrhizal fungi and organic manure have synergistic effects on Trifolium repens in Cd-contaminated sterilized soil but not in natural soil. Appl. Soil Ecol. 2020;149:103485. https://doi.org/10.1016/j.apsoil.2019.103485.
35. Artursson V, Finlay RD, and Jansson JK. Interactions between arbuscular mycorrhizal fungi and bacteria and their potential for stimulating plant growth. Environ. Microbiol. 2006;8(1):1-10. https://doi.org/10.1111/j.1462-2920.2005.00942.x.
36. Fester T, Maier W, and Strack D. Accumulation of secondary compounds in barley and wheat roots in response to inoculation with an arbuscular mycorrhizal fungus and co-inoculation with rhizosphere bacteria. Mycorrhiza. 1999;8(5):241-246. https://doi.org/doi:10.1007/s005720050240
37. Battini F, Cristani C, Giovannetti M, and Agnolucci M. Multifunctionality and diversity of culturable bacterial communities strictly associated with spores of the plant beneficial symbiont Rhizophagus intraradices. Microbiol. Res. 2016;183:68-79. https://doi.org/10.1016/j.micres.2015.11.012.
38. Garbaye J. Tansley review no. 76 helper bacteria: a new dimension to the mycorrhizal symbiosis. New Phytol. 1994;128(2):197-210. https://doi.org/10.1111/j.1469-8137.1994.tb04003.x
39. Frey-Klett P, Garbaye J, and Tarkka M. The mycorrhiza helper bacteria revisited. New Phytol. 2007;176(1):22-36. https://doi.org/10.1111/j.1469-8137.2007.02191.x.
40. Srivastava R, Khalid A, Singh US, and Sharma AK. Evaluation of arbuscular mycorrhizal fungus, fluorescent Pseudomonas and Trichoderma harzianum formulation against Fusarium oxysporum f. sp. lycopersici for the management of tomato wilt. Biol. Control. 2010;53(1):24-31. https://doi.org/10.1016/j.biocontrol.2009.11.012.
41. Azcón-Aguilar C and Barea JM. Arbuscular mycorrhizas and biological control of soil-borne plant pathogens–an overview of the mechanisms involved. Mycorrhiza. 1997;6(6):457-464. https://doi.org/10.1007/s005720050147
42. Doumbou CL, Hamby Salove MK, Crawford DL, and Beaulieu C. Actinomycetes, promising tools to control plant diseases and to promote plant growth. Phytoprotection. 2005;82(3):85-102. https://doi.org/10.7202/706219ar.
43. Tan KH, *Soil sampling, preparation, and analysis: CRC press*. 2005.
44. Giovannetti M and Mosse B. An evaluation of techniques for measuring vesicular-arbuscular infection in roots. New Phytol. 1980;84(3):489-500. https://doi.org/10.1111/j.1469-8137.1980.tb04556.x. .
45. Bao SD, *Soil and agricultural chemistry analysis*. 2000, China agriculture press, Beijing.
46. Hoeksema JD, Chaudhary VB, Gehring CA, Johnson NC, Karst J, Koide RT, et al. A meta-analysis of context-dependency in plant response to inoculation with mycorrhizal fungi. Ecol. Lett. 2010;13(3):394-407. https://doi.org/10.1111/j.1461-0248.2009.01430.x.
47. Hedges LV, Gurevitch J, and Curtis PS. The Meta-Analysis of Response Ratios in Experimental Ecology. Ecology.

1999;80(4):1150-1156. https://doi.org/10.2307/177062

48. Bona E, Cantamessa S, Massa N, Manassero P, Marsano F, Copetta A, et al. Arbuscular mycorrhizal fungi and plant growth-promoting pseudomonads improve yield, quality and nutritional value of tomato: a field study. Mycorrhiza. 2017;27(1):1-11. https://doi.org/10.1007/s00572-016-0727-y.
49. Zhang F, Li Q, Yerger EH, Chen X, Shi Q, and Wan F. AM fungi facilitate the competitive growth of two invasive plant species, Ambrosia artemisiifolia and Bidens pilosa. Mycorrhiza. 2018;28(8):703-715. https://doi.org/10.1007/s00572-018-0866-4.
50. Allen MF. Linking water and nutrients through the vadose zone: a fungal interface between the soil and plant systems. J. Arid Land. 2011;3(3):155-163. https://doi.org/10.3724/sp.J.1227.2011.00155.
51. Smith SE and Smith FA. Roles of Arbuscular Mycorrhizas in Plant Nutrition and Growth: New Paradigms from Cellular to Ecosystem Scales. Annu. Rev. Plant Biol. 2011;62(1):227-250. https://doi.org/10.1146/annurev-arplant-042110-103846.
52. Lugtenberg BJJ, Malfanova N, Kamilova F, and Berg G. Plant Growth Promotion by Microbes. Molecular Microbial Ecology of the Rhizosphere 2013;2:559-573. https://doi.org/doi:10.1002/9781118297674.ch53
53. Leff JW, Jones SE, Prober SM, Barberan A, Borer ET, Firn JL, et al. Consistent responses of soil microbial communities to elevated nutrient inputs in grasslands across the globe. Proceedling of the National Academy of Science. 2015;112(35):10967-10972. https://doi.org/10.1073/pnas.1508382112.
54. Tinker PB and Nye PH, *Solute movement in the rhizosphere*. 2000: Oxford University Press.
55. Leigh J, Hodge A, and Fitter AH. Arbuscular mycorrhizal fungi can transfer substantial amounts of nitrogen to their host plant from organic material. New Phytol. 2009;181(1):199-207. https://doi.org/10.1111/j.1469-8137.2008.02630.x.
56. Shao YD, Hu XC, Wu QS, Yang TY, Srivastava AK, Zhang DJ, et al. Mycorrhizas promote P acquisition of tea plants through changes in root morphology and P transporter gene expression. S. Afr. J. Bot. 2021;137:455-462. https://doi.org/10.1016/j.sajb.2020.11.028.
57. Tawaraya K, Naito M, and Wagatsuma T. Solubilization of Insoluble Inorganic Phosphate by Hyphal Exudates of Arbuscular Mycorrhizal Fungi. J. Plant Nutr. 2006;29(4):657-665. https://doi.org/10.1080/01904160600564428.
58. Wu F, Li Z, Lin Y, and Zhang L. Effects of *Funneliformis mosseae* on the utilization of organic phosphorus in Camellia oleifera Abel. Can. J. Microbiol. 2021;67(5):349-357. https://doi.org/10.1139/cjm-2020-0227.
59. Cappellazzo G, Lanfranco L, Fitz M, Wipf D, and Bonfante P. Characterization of an amino acid permease from the endomycorrhizal fungus *Glomus mosseae.* Plant Physiol. 2008;147(1):429-37. https://doi.org/10.1104/pp.108.117820.
60. Hodge A, Campbell CD, and Fitter AH. An arbuscular mycorrhizal fungus accelerates decomposition and acquires nitrogen directly from organic material. Nature. 2001;413:297-299. https://doi.org/10.1038/35095041
61. Elbon A and Whalen JK. Phosphorus supply to vegetable crops from arbuscular mycorrhizal fungi: a review. Biol. Agric. Hortic. 2014;31(2):73-90. https://doi.org/10.1080/01448765.2014.966147.
62. Zhang LX, Bai YF, and Han XG. Differential Responses of N:P Stoichiometry of Leymus chinensis and Carex korshinskyi to N Additions in a Steppe Ecosystem in Nei Mongol. Acta Bot. Sin. . 2004;46:259-270. https://doi.org/10.1300/J079v30n03_01.
63. Koerselman, Willem;, and Meuleman AFM. The vegetation N:P ratio: a new tool to detect the nature of nutrient limitation. J. App. Ecol. 1996;33(6):1441-1450. https://doi.org/doi:10.2307/2404783

64. Shen K, Cornelissen JHC, Wang Y, Wu C, He Y, Ou J, et al. AM fungi alleviate phosphorus limitation and enhance nutrient competitiveness of invasive plants via mycorrhizal networks in karst areas. Front. Ecol. Evol. 2020;8. https://doi.org/10.3389/fevo.2020.00125.
65. Bever JD. Soil community feedback and the coexistence of competitors: conceptual frameworks and empirical tests. New Phytol. 2003;157(3):465-473. https://doi.org/10.2307/1514052
66. Van der Heijden MG, Boller T, Wiemken A, and Sanders IR. Different arbuscular mycorrhizal fungal species are potential determinants of plant community structure. Ecology. 1998;79(6):2082-2091.
67. Liu R and Wang F. Selection of appropriate host plants used in trap culture of arbuscular mycorrhizal fungi. Mycorrhiza. 2003;13(3):123-127. https://doi.org/10.1007/s00572-002-0207-4.
68. Danuso F, Zanin G, and Sartorato I. A modelling approach for evaluating phenology and adaptation of two congeneric weeds (Bidens frondosa and Bidens tripartita). Ecol. Model. 2012;243:33-41. https://doi.org/10.1016/j.ecolmodel.2012.06.009
69. Bartolome AP, Villaseñor IM, and Yang WC. Bidens pilosa L.(Asteraceae): botanical properties, traditional uses, phytochemistry, and pharmacology. Evid-Based Compl. Alt. 2013;2013. https://doi.org/10.1155/2013/340215.
70. Wilson GW, Rice CW, Rillig MC, Springer A, and Hartnett DC. Soil aggregation and carbon sequestration are tightly correlated with the abundance of arbuscular mycorrhizal fungi: results from long-term field experiments. Ecol. Lett. 2009;12(5):452-61. https://doi.org/10.1111/j.1461-0248.2009.01303.x.
71. Giovannini L, Palla M, Agnolucci M, Avio L, Sbrana C, Turrini A, et al. Arbuscular mycorrhizal fungi and associated microbiota as plant biostimulants: research strategies for the selection of the best performing inocula. Agronomy-Basel. 2020;10(1):106. https://doi.org/10.3390/agronomy10010106.
72. Vázquez MM, César S, Azcón R, and Barea JM. Interactions between arbuscular mycorrhizal fungi and other microbial inoculants (Azospirillum, Pseudomonas, Trichoderma) and their effects on microbial population and enzyme activities in the rhizosphere of maize plants. Applied Soil Ecology. 2000;15(3):261-272. https://doi.org/doi:10.1016/s0929-1393(00)00075-5
73. Larimer AL, Clay K, and Bever JD. Synergism and context dependency of interactions between arbuscular mycorrhizal fungi and rhizobia with a prairie legume. Ecological. 2014;95(4):1045-1054. https://doi.org/10.1890/13-0025.1
74. Diagne N, Baudoin E, Svistoonoff S, Ouattara C, Diouf D, Kane A, et al. Effect of native and allochthonous arbuscular mycorrhizal fungi on Casuarina equisetifolia growth and its root bacterial community. Arid Land Res. Manage. 2017;32(2):212-228. https://doi.org/10.1080/15324982.2017.1406413.
75. Mortimer PE, Le Roux MR, Pérez-Fernández MA, Benedito VA, Kleinert A, Xu J, et al. The dual symbiosis between arbuscular mycorrhiza and nitrogen fixing bacteria benefits the growth and nutrition of the woody invasive legume Acacia cyclops under nutrient limiting conditions. Plant Soil. 2012;366(1-2):229-241. https://doi.org/10.1007/s11104-012-1421-2.
76. Linderman RG, *Vesicular-arbuscular mycorrhizal (VAM) fungi*, in *In: Carroll GC, Tudzynski P (eds) The Mycota*. 1997, Springer-Verlag Berlin, Germany.
77. Miransari M. Interactions between arbuscular mycorrhizal fungi and soil bacteria. Appl. Microbiol. Biotechnol. 2011;89(4):917-930. https://doi.org/10.1007/s00253-010-3004-6.
78. Rousk J, Demoling LA, Bahr A, and Baath E. Examining the fungal and bacterial niche overlap using selective inhibitors in soil. FEMS Microbiol. Ecol. 2008;63(3):350-358. https://doi.org/10.1111/j.1574-6941.2008.00440.x.
79. de Boer W, Verheggen P, Klein Gunnewiek PJ, Kowalchuk GA, and van Veen JA. Microbial community

composition affects soil fungistasis. Appl. Environ. Microbiol. 2003;69(2):835-44. https://doi.org/10.1128/AEM.69.2.835-844.2003.

80. Li X, Garbeva P, Liu X, Klein Gunnewiek PJA, Clocchiatti A, Hundscheid MPJ, et al. Volatile-mediated antagonism of soil bacterial communities against fungi. Environ. Microbiol. 2020;22(3):1025-1035. https://doi.org/10.1111/1462-2920.14808.
81. Leigh J, Fitter AH, and Hodge A. Growth and symbiotic effectiveness of an arbuscular mycorrhizal fungus in organic matter in competition with soil bacteria. FEMS Microbiol. Ecol. 2011;76(3):428-438. https://doi.org/10.1111/j.1574-6941.2011.01066.x.
82. Niwa R, Koyama T, Sato T, Adachi K, Tawaraya K, Sato S, et al. Dissection of niche competition between introduced and indigenous arbuscular mycorrhizal fungi with respect to soybean yield responses. Sci. Rep-UK. 2018;8(1):7419. https://doi.org/10.1038/s41598-018-25701-4.
83. Biró B, Köves-Péchy K, Takács IVT, Eggenberger P, and Strasser. RJ. Interrelations between Azospirillum and Rhizobium nitrogen-fixers and arbuscular mycorrhizal fungi in the rhizosphere of alfalfa in sterile, AMF-free or normal soil conditions. Appl. Soil Ecol. 2000;15(2):159-168. https://doi.org/10.1016/s0929-1393(00)00092-5

Legends

Fig. 1 The biomass (A) and its response ratio *$lnR_{Biomass}$* (B) of four plant species through the different microbial treatments. Abbreviations: *S. v* = *Setaria viridis*; *A. h* = *Arthraxon hispidus*; *B. p* = *Bidens pilosa*; *B. t* = *Bidens tripartita*; *AMF* = the mycorrhizal fungi soil by AM fungi inoculation; *AMI* = the combining soil by AM fungi with indigenous microorganism; *CK* = the sterilized soil as the control by removing microorganism; *AME* = AM fungi effect; *AIE* = interactive effect related to AM fungi interacting with indigenous microbes; I*ME* = indigenous microbial effect related to AM fungi. The different lowercase letters (a, b, c, d) indicate significant differences between species under *AMF*, *AMI,* and *CK* treatments, respectively; The different lowercase letters (x, y, z) indicate significant differences between *AMF*, *AMI,* and *CK* treatments for the same species ($P < 0.05$).

Fig. 2 N accumulation (A) and response ratio *lnR_N* (B) of four plant species through the different microbial treatments. The meanings of abbreviations (*S. v*, *A. h*, *B. p* and *B. t*; *AMF*, *AMI,* and *CK*; *AME*, *AIE* and I*ME*) and the lowercase letters (a, b, c, d; x, y, z) are the same as in Fig 1.

Fig. 3 P accumulation (A) and response ratio *lnR_P* (B) of four plant species through the different microbial treatments. The meanings of abbreviations (*S. v*, *A. h*, *B. p* and *B. t*; *AMF*, *AMI,* and *CK*; *AME*, *AIE* and I*ME*) and the lowercase letters (a, b, c, d; x, y, z) are the same as in Fig 1.

Fig. 4 N/P ratio (A) and response ratio *$lnR_{N/P}$* (B) of four plant species through the different microbial treatments. The meanings of abbreviations (*S. v*, *A. h*, *B. p* and *B. t*; *AMF*, *AMI,* and *CK*; *AME*, *AIE* and I*ME*) and the lowercase letters (a, b, c, d; x, y, z) are the same as in Fig 1.

Table 1 the mycorrhizal colonization rates of *Setaria viridis, Arthraxon hispidus, Bidens pilosa,* and *Bidens tripartita*

Treatment	Mycorrhizal colonization			
	S.viridis	***A.hispidus***	***B.pilosa***	***B.tripartita***
AMI	20.40±0.68cx	48.60±1.17bx	65.00±1.48ax	67.40±1.29ax
AMF	18.80±1.43cx	46.40±1.78bx	62.20±1.46ax	64.60±1.17ax

The different lowercase letters (a, b, c, d) indicate significant differences between plant species of *Setaria viridis*, *Arthraxon hispidus*, *Bidens pilosa*, and *Bidens tripartita* at the 0.05 level; The different lowercase letters (x, y) indicate significant differences between *AMF*, *AMI*, treatments under the same plant.

Table 2 Two-Way ANOVAs for the effects of plant species (S. *viridis vs. A. hispidus vs. B. pilosa vs. B. tripartita*) and soil microbial condition (*AMF vs. AMI vs. CK*) on the biomass, the N accumulation, and their response ratio (ln*R*).

Factors	*df*	Biomass		Response ratio of biomass ($\ln R_{Biomass}$)		N accumulation		Response ratio of N ($\ln R_N$)	
		F	*P*	*F*	*P*	*F*	*P*	*F*	*P*
Ms	2	124.072	0.000***	542.979	0.000***	117.557	0.000***	416.907	0.000***
Ps	3	34.012	0.000***	59.966	0.000***	21.133	0.000***	54.208	0.000***
Ms×Ps	6	27.106	0.000***	33.041	0.000***	13.492	0.000***	24.766	0.000***

Abbreviations: Ms =Soil microbial condition treatments; Ps = Plant species; * or ** or *** indicates a significant difference in $P < 0.05$ or $P < 0.01$ or $P < 0.001$.

Table 3 Two-Way ANOVAs for the effects of plant species (*S. viridis vs. A. hispidus vs. B. pilosa vs. B. tripartita*) and soil microbial condition (*AMF vs. AMI vs. CK*) on the P accumulation, the N/P ratio, and their response ratio (ln*R*).

Factors	*df*	P accumulation		Response ratio of P ($\ln R_P$)		N/P ratio		Response ratio of N/P ($\ln R_{N/P}$)	
		F	*P*	*F*	*P*	*F*	*P*	*F*	*P*
Ms	2	102.158	0.000***	394.863	0.000***	8.263	0.000***	10.936	0.000***
Ps	3	15.069	0.000***	24.168	0.000***	20.876	0.000***	40.158	0.000***
Ms×Ps	6	12.138	0.000***	44.834	0.000***	9.569	0.000***	12.175	0.000***

Abbreviations: Ms =Soil microbial condition treatments; Ps = Plant species; * or ** or *** indicates a significant difference in $P < 0.05$ or $P < 0.01$ or $P < 0.001$.

Click here to access/download;Figure;Fig 1.tif

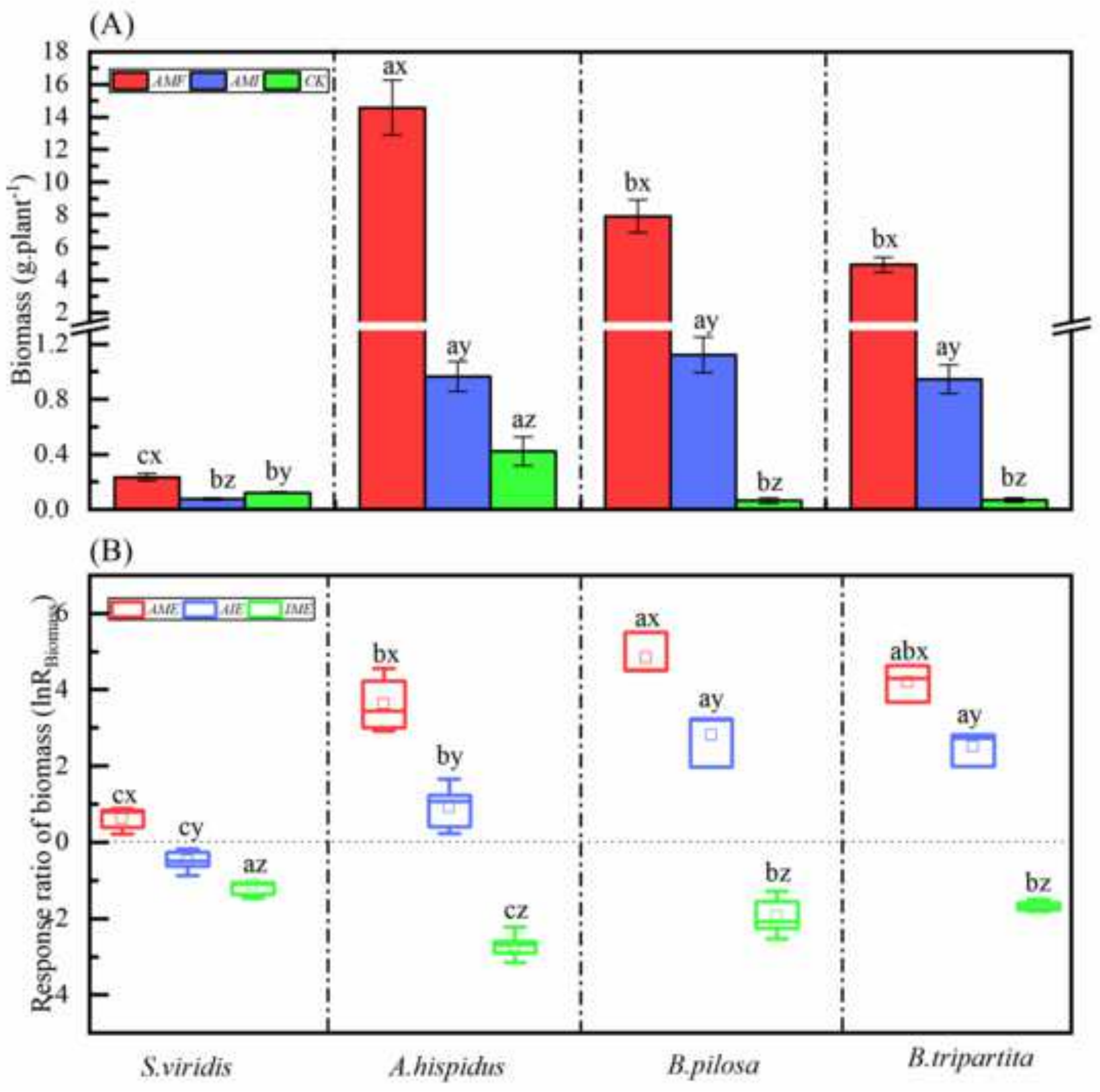

Figure

Click here to access/download;Figure;Fig 2.tif

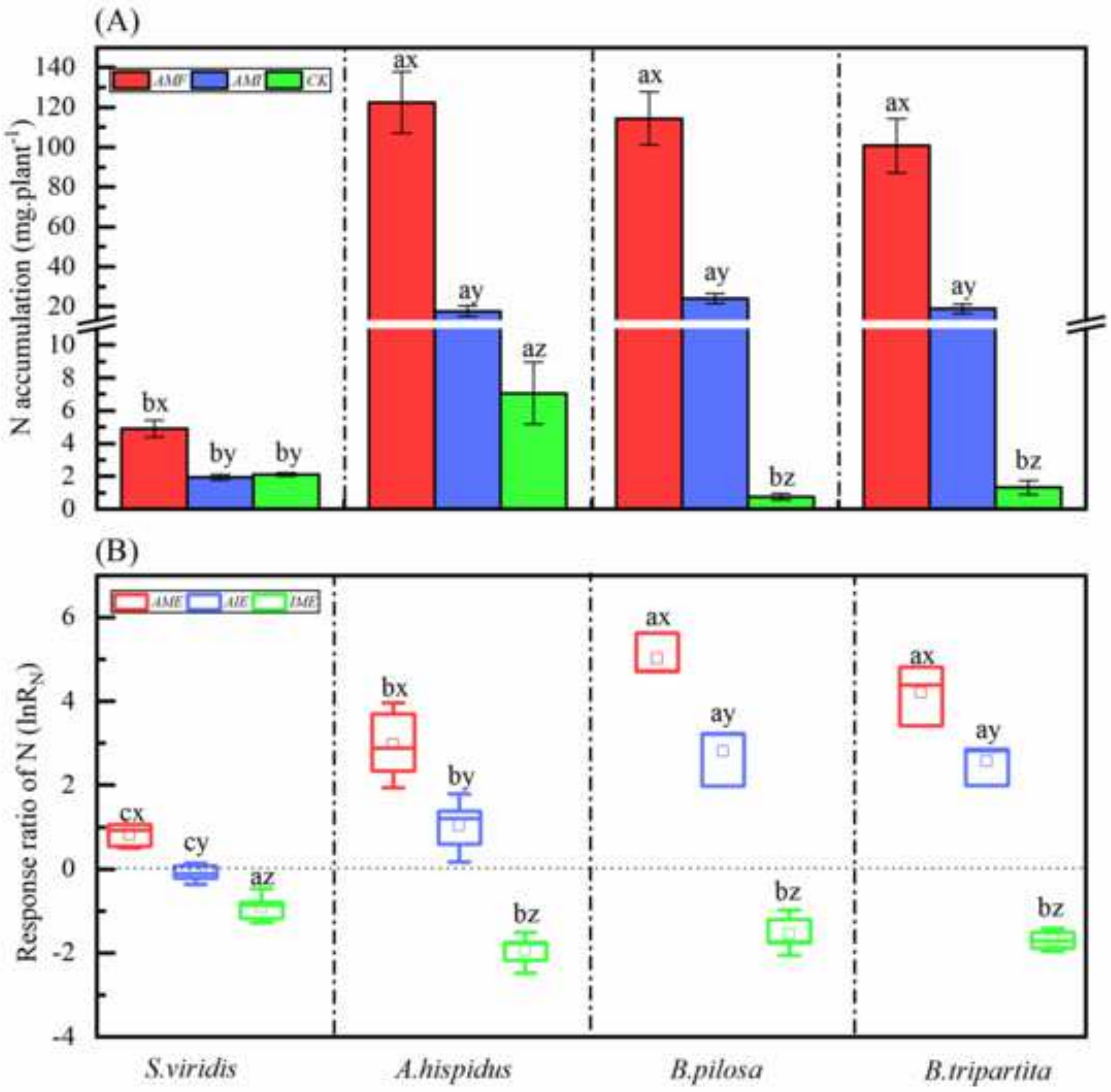

Click here to access/download;Figure;Fig 3.tif

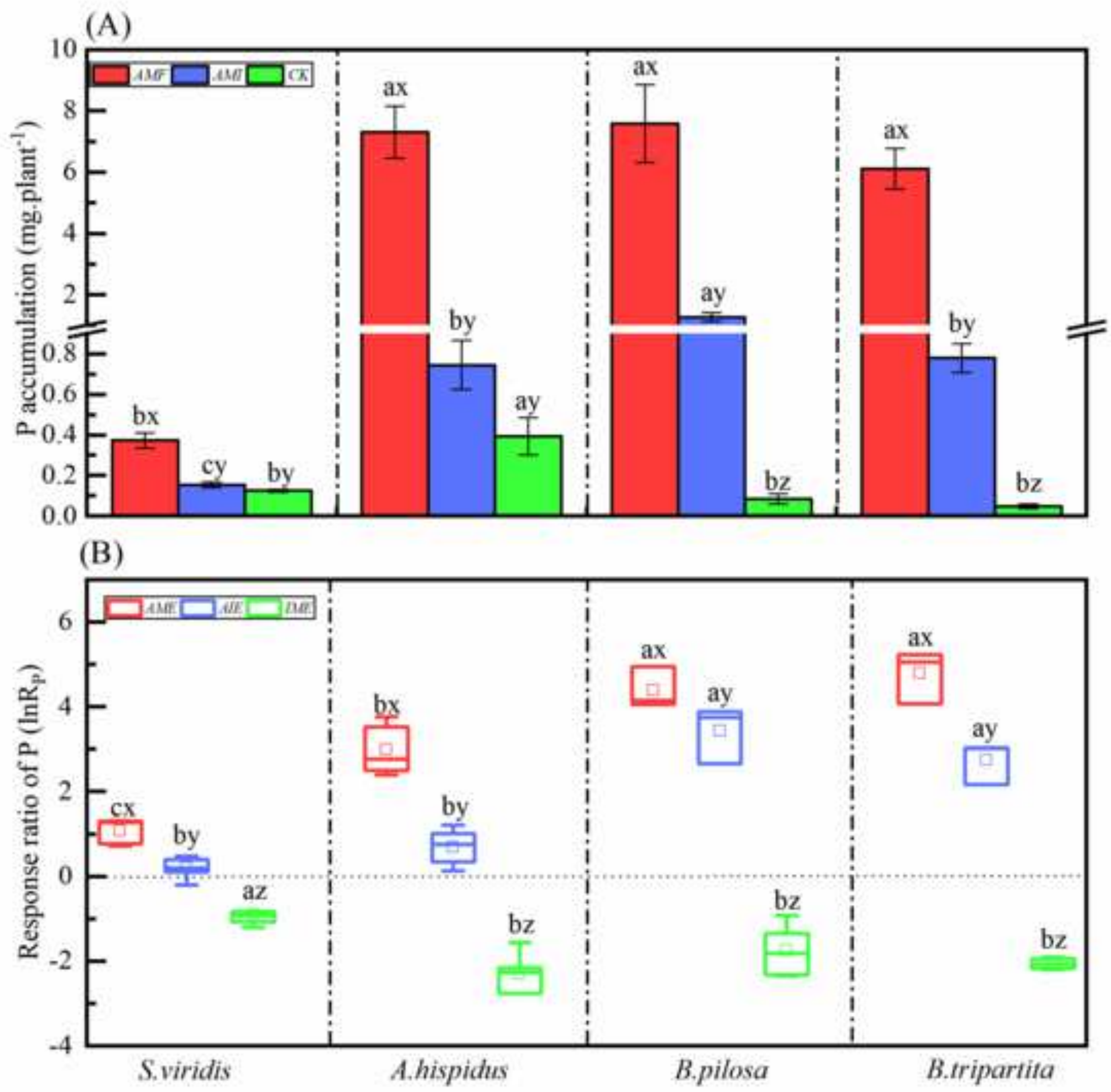

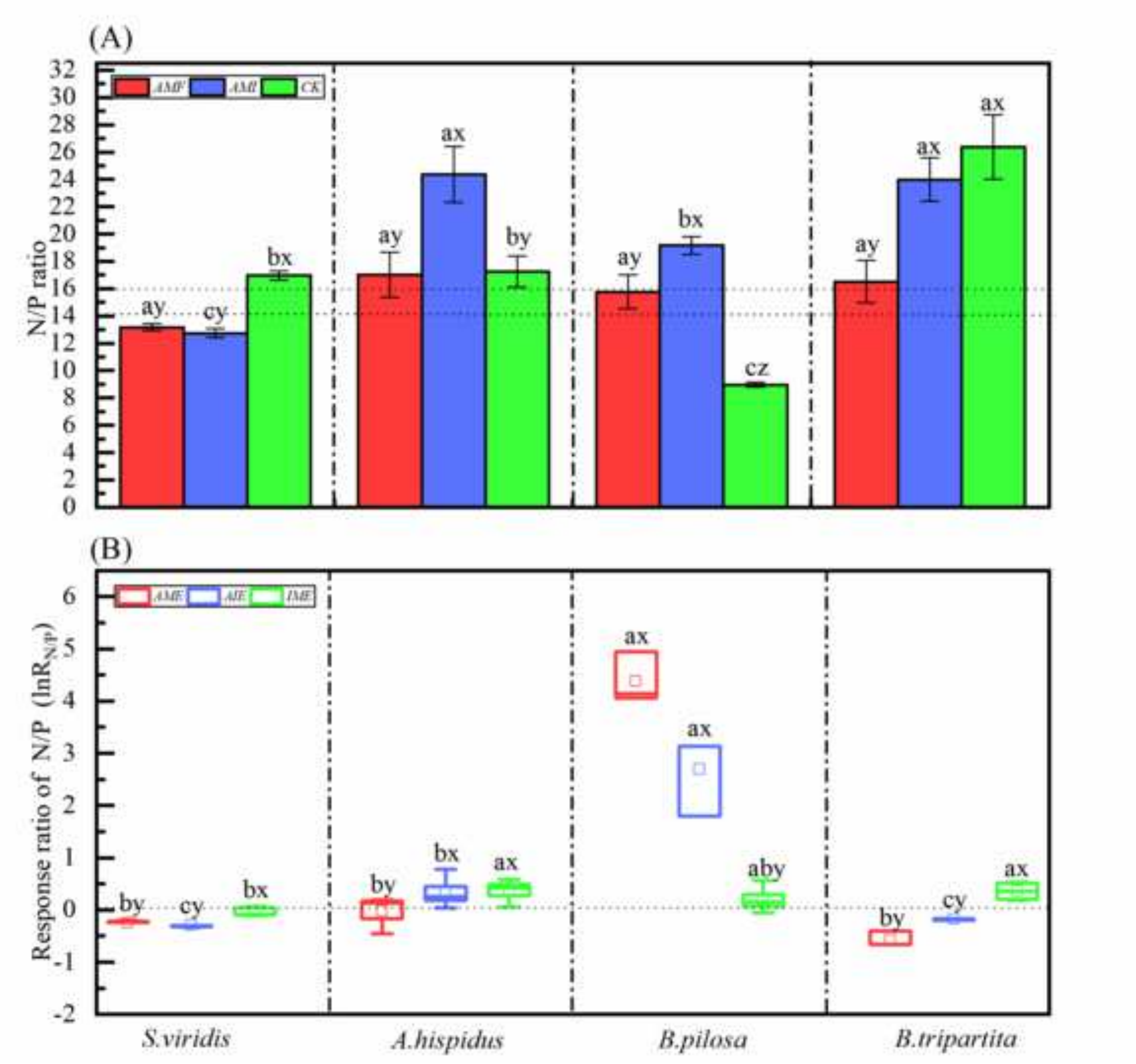
(A)
N/P ratio
AMF
AMI
CK
(B)
Response ratio of N/P (lnR$_{N/P}$)
AME
AIE
IME
S.viridis
A.hispidus
B.pilosa
B.tripartita
