# Supplementary material for: Indigenous microorganisms offset the benefits of growth and nutrition regulated by inoculated arbuscular mycorrhizal fungi for four pioneer herbs in karst soil
Source: PLoS One. 2022 Apr 25;17(4):e0266526. doi: 10.1371/journal.pone.0266526 (PMC9037916; doi:10.1371/journal.pone.0266526)
Supplement: Attachment — Submitted filename: Response to Reviewers.docx [file pone.0266526.s003.docx]

**Journal Requirements**

**Q1:** Please review your reference list to ensure that it is complete and correct. If you have cited papers that have been retracted, please include the rationale for doing so in the manuscript text, or remove these references and replace them with relevant current references. Any changes to the reference list should be mentioned in the rebuttal letter that accompanies your revised manuscript. If you need to cite a retracted article, indicate the article’s retracted status in the References list and also include a citation and full reference for the retraction notice.

**RESPONSE:** Thank you for your comments. According to the suggestions of Reviewer #1, we refined the discussion part and deleted five references [55], [56], [57] [69], [70] in the original manuscript, as follows:

[55] Tinker PB and Nye PH, Solute movement in the rhizosphere. 2000: Oxford University Press.

[56] Leigh J, Hodge A, and Fitter AH. Arbuscular mycorrhizal fungi can transfer substantial amounts of nitrogen to their host plant from organic material. New Phytol. 2009;181(1):199-207. https://doi.org/10.1111/j.1469- 556

8137.2008.02630.x. 557

[57] Shao YD, Hu XC, Wu QS, Yang TY, Srivastava AK, Zhang DJ, et al. Mycorrhizas promote P acquisition of tea plants through changes in root morphology and P transporter gene expression. S. Afr. J. Bot. 2021;137:455-462.

<https://doi.org/10.1016/j.sajb.2020.11.028>.

[69] Danuso F, Zanin G, and Sartorato I. A modelling approach for evaluating phenology and adaptation of two congeneric weeds (Bidens frondosa and Bidens tripartita). Ecol. Model. 2012;243:33-41. https://doi.org/10.1016/j.ecolmodel.2012.06.009 589

[70] Bartolome AP, Villaseñor IM, and Yang WC. Bidens pilosa L.(Asteraceae): botanical properties, traditional uses, phytochemistry, and pharmacology. Evid-Based Compl. Alt. 2013;2013. https://doi.org/10.1155/2013/340215.

**Reviewer #1:**

**Q1:** Line 37-39. What is the purpose to compare the growth status of the species in this experiment? Is it essential for this topic?

**RESPONSE:** Thank you for your comments. We checked and agreed with your view that it is not essential for this topic in the Abstract section, so we deleted Line 37-39 in the original manuscript.

**Q2:** Line 93-94. What were the evidences to support this hypothesis before this research was conducted?

**RESPONSE:** Thank you for your comments and questions.

In the revised version: (1) we revised the original summary between AM fungi and indigenous microorganisms to “Thus, the cooperation and competition between AM fungi and indigenous microorganisms are ineluctability in karst soil” See Line80-81 of the revision.

(2) we added the two sentences about previous studies as evidences to support the hypothesis, see Line 91-92 and Line 93-95 of the revision.

**Q3:** Please add sub-headlines for the Discussion part. It is not clear what is the central topic for each paragraph. Still，there are too many discussions on the roles of AMF on plant growth, which were not the central topic of this study.

**RESPONSE:** Thank you for your good suggestions. We added two sub-headlines for the Discussion part; see Line 297 and Line 340-341 of the revision. In addition, we further refined the Discussion section, please see the new version.

**Q4:** Line 396-399. The first argument is self-contradictory with the following statement.

**RESPONSE:** Many thanks for your comments and questions. We have checked and revised carefully, in order to express clearer, we modified “while the indigenous microorganisms offset the benefits of AM fungi foe host plants” to “However, the benefits from interaction were lower than benefits from AM, indicating that the indigenous microorganisms offset the benefits of AM fungi for host plants”. See Line 390 of the revision.

**Q5:** Line 399-401. What is the significance of this finding?

**RESPONSE:** Thanks a lot for your comments. We checked and agreed with your view that it is not essential for this topic in this manuscript, and we thought it is not the significance of this finding in this manuscript. Thus, we deleted Line 399-401 in the original manuscript.

**Q6:** Some grammar mistakes and English expressions are corrected in the tracked PDF.

**RESPONSE:** Thank you for your good suggestions. We further checked the language and refined expression, and modified some grammar mistakes and English expressions in the whole manuscript in the new revision according to your suggestions.
