# Supplementary material for: Indigenous microorganisms offset the benefits of growth and nutrition regulated by inoculated arbuscular mycorrhizal fungi for four pioneer herbs in karst soil
Source: PLoS One. 2022 Apr 25;17(4):e0266526. doi: 10.1371/journal.pone.0266526 (PMC9037916; doi:10.1371/journal.pone.0266526)
Supplement: Attachment — Submitted filename: Response to Reviewers.docx [file pone.0266526.s004.docx]

**Response**

Dear editor,

Many thanks for the comments on our revision to the very positive original reviewer. We appreciate that our manuscript still needed further work, which entitled “Indigenous microorganisms offset the benefits of growth and nutrition regulated by inoculated arbuscular mycorrhizal fungi for four pioneer herbs in karst soil” (PONE-D-21-40761R2). We revised this sentence of “the benefits form interaction” being “the benefits from interaction” according to Journal Requirements. In addition, we checked the full text and corrected all in grammar and style through the marked in blue fonts in this new version. We hope it will meet with your approval, and also hope the revision can meet the quality standard for publication in *PLoS ONE***.** The main corrections in the paper and the response to the reviewers’ comments are as follows by **Journal Requirements**

All best regards

Yours sincerely,

Yan Sun, Yuejun He

Behalf of all authors

**Journal Requirements**

**Q1:** The revised version has been improved a lot. All the comments have been addressed. But the authors still need to polish the language and revise the language errors.
For example:
Line 390 : “the benefits form interaction” should be “the benefits from interaction”

**RESPONSE:** Thank you for your comments and questions. We revised this sentence of “the benefits form interaction” being “the benefits from interaction”, see Line 383 of the revision. In addition, we further checked and modified the language and revised the language errors. Please see the new version.
